# A Systematic Review of the Need for Guideline Recommendations; Slow Tapering vs. Maintenance Dose in Long-Term Antipsychotic Treatment: 2022

**DOI:** 10.7759/cureus.34746

**Published:** 2023-02-07

**Authors:** Shanthi Potla, Yousif Al Qabandi, Savitri Aninditha Nandula, Chinmayi Sree Boddepalli, Sai Dheeraj Gutlapalli, Vamsi Krishna Lavu, Rana Abdelwahab Mohamed Abdelwahab, Ruimin Huang, Pousette Hamid

**Affiliations:** 1 Psychiatry and Behavioral Sciences, California Institute of Behavioral Neurosciences & Psychology, Fairfield, USA; 2 Internal Medicine, California Institute of Behavioral Neurosciences & Psychology, Fairfield, USA; 3 Dermatology, California Institute of Behavioral Neurosciences & Psychology, Fairfield, USA; 4 Neurology, California Institute of Behavioral Neurosciences & Psychology, Fairfield, USA

**Keywords:** antipsychotic, maintenance dose, psychosis, relapse, schizophrenia and other psychotic disorders, tapering

## Abstract

The act of discontinuing the antipsychotic medication may be directly associated with relapse. This relationship might be due to adaptations that continue to exist after treatment is stopped, such as dopaminergic hypersensitivity. Therefore, more progressive weaning off antipsychotic medication may help reduce the likelihood of relapse when the medication is stopped. As there is a need to gradually reduce or stop using antipsychotic medication, our team tried to conduct a more in-depth search to give further answers to the suggested recommendations. Around 192 articles were gathered for our research, but we could only narrow our search to 27, which were further filtered, and eight were used. We went through all of the pertinent information available until May 2022 and reviewed it to determine the risks associated with prolonged antipsychotic usage and abrupt cessation in the psychotic spectrum of diseases. PubMed, Google Scholar, and Psychiatry Online were the databases used, and the keywords that were looked for and utilized were antipsychotics, tapering, relapse, maintenance dosage, schizophrenia, and psychosis. The recurrence incidence was high in patients in whom antipsychotics were stopped and in whom the dosage was quickly lowered. Patients who were gradually weaned off their antipsychotic medication and kept on the lowest effective dose had a much lower risk of experiencing a relapse. We suggest more studies, including randomized clinical trials and monitoring, considering the enhancement of guidelines for the total cessation of antipsychotic medication use.

## Introduction and background

Antipsychotic medicine reduces the occurrence of psychotic symptoms and the chance of relapse [1]. Relapse prevention is critical for antipsychotic drug treatment because it improves patient outcomes and decreases hospitalizations [2]. However, relapse has serious consequences, including functional disability and financial burden [2]. Furthermore, it may cause biological changes, increasing the risk of future treatment refractoriness [3]. It is also evident that patients frequently discontinue or seek to discontinue medication following remission, and clinicians are frequently asked primarily about the danger of relapse [3]. After a successful long-term treatment, the medication may be weaned off or kept on a maintenance dose. 

Moreover, most patients often want to discontinue antipsychotic drugs due to their side effects [4]. Relapse rates remained the dominant outcome criterion in assessing the efficacy of antipsychotic treatment methods [4]. Though outcome research has shifted attention to customers' opinions and recovery, antipsychotic effectiveness is still almost entirely determined by symptom severity and relapse rates [5].

Psychosis is a spectrum of mental health disorders primarily treated with medication-blocking dopaminergic receptors. There are four major dopaminergic pathways, among which blocking D2-receptors (dopamine2) in the mesolimbic pathway treats positive symptoms, and releasing dopamine in the mesocortical pathway alleviates negative symptoms [6]. Dopamine hypersensitization produced by long-term antipsychotic use was the cause of relapses [5]. The anticipated dopamine hypersensitivity caused by dopamine D2 blockage was supposed to cause not just rebound psychosis but also treatment resistance and tolerance to antipsychotics, causing these drugs to lose their effectiveness [5]. Upon discontinuation, high recurrence rates result from sudden discontinuation rather than progressive tapering [5].

Although evidence of antipsychotic medication's benefits in short-term treatment exists, the need for and effectiveness of preventative long-term antipsychotics in all people with schizophrenia is currently being contested [7]. In addition, with multiple antipsychotic discontinuation studies underway, it is still unclear what percentage of patients may be able to stay well without antipsychotics [7]. However, some estimates suggest it could be as high as 40% [7]. This research examines what is known about maintaining vs. terminating antipsychotics. We assess the result among patients on the lowest dose of antipsychotic medication after following a tapering procedure that includes full spectrum relapse, hospitalization, withdrawal syndromes, and guidelines for maintaining and stopping antipsychotics while minimizing the risk of recurrence [7]. Based on the relapse's severity, the quest of this research is to determine whether to continue patients on the lowest tapering dose, discontinue, or discontinue after a more prolonged maintenance dose.

## Review

Methodology

**Figure 1:**
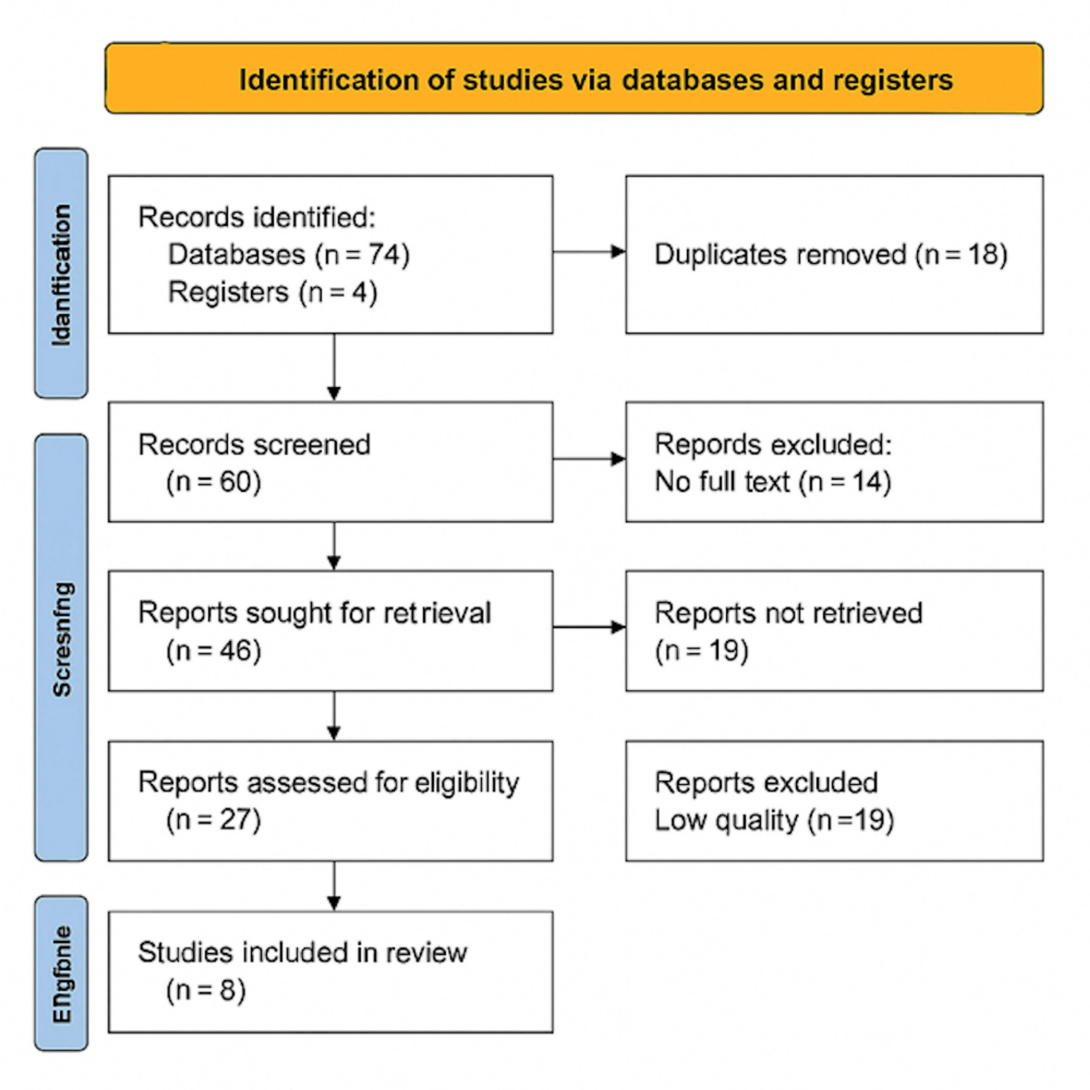
PRISMA flow diagram

We conducted our systematic review (Figure 1) using Preferred Reporting Items for Systematic Reviews and Meta-Analyses (PRISMA) guidelines:

Database

We started our research on April 2^nd^, 2022, using online libraries as our database. We searched PubMed, PubMed Central, Google Scholar, and Cochrane Library for our data collection.

Search strategy

We included studies related to tapering/medications and schizophrenia/psychosis. Our keywords and medical subject heading (MeSH) search strategies included tapering, mental health, protocol, antipsychotics, relapse, schizophrenia, and psychosis. We included the results for each search in Table 1.

**Table 1:** Initial search results data

Keyword/ MeSH keyword	Database	Search result number
Antipsychotic	PubMed Central	57386
	PubMed	26442
	Google Scholar	560000
	Psychiatry Online	10559
tapering	PubMed Central	37651
	PubMed	24660
	Google Scholar	460000
	Psychiatry Online	1760
relapse	PubMed Central	765923
	PubMed	686597
	Google Scholar	2150000
	Psychiatry Online	7923
Antipsychotics, tapering	PubMed Central	941
	PubMed	309
	Google Scholar	18400
	Psychiatry Online	675
Schizophrenia	PubMed Central	192343
	PubMed	155597
	Google Scholar	2390000
	Psychiatry Online	31281
Antipsychotics, tapering, relapse	PubMed Central	509
	PubMed	60
	Google Scholar	9190
	Psychiatry Online	258
Maintenance dose, relapse	PubMed Central	192343
	PubMed	155597
	Google Scholar	2390000
	Psychiatry Online	31281
Maintenance dose, schizophrenia, psychosis	PubMed Central	7571
	PubMed	109
	Google Scholar	62400
	Psychiatry Online	3058
Maintenance dose, relapse, Schizophrenia, psychosis	PubMed Central	3597
	PubMed	43
	Google Scholar	37500
	Psychiatry Online	1137

Initial search results

Table 1 represents the search result numbers that we found for each keyword or MeSH (Medical Subject Heading) keyword.

Inclusion criteria

We chose peer-reviewed articles and studies published in English in the previous five years. We only included human studies in the categories of systematic reviews, traditional reviews, meta-analyses, and randomized trials. All information gathered is ethical and legal.

Exclusion criteria

Gray literature and animal studies were excluded. We also excluded studies conducted in the previous five years.

Quality assessment tools

For systematic reviews and meta-analysis, we used the AMSTAR questionnaire, Cochrane risk bias assessment tools for clinical trials, the New Castle-Ottawa questionnaire for observational studies, and a scale for the quality assessment of narrative review articles (SANRA) scale for traditional reviews. We excluded studies with poor quality.

Data collection

Data was collected from the final articles after a quality assessment based on Table 1 results.

Result

We chose 78 studies after screening the titles and reviewing them after searching the database. We had 60 articles after screening them and removing 18 duplicates (Figure 1); we found 14 abstracts without full articles and excluded them from our final list. We excluded 19 articles after reviewing the full articles because they were not relevant to our study. For the remaining 27 articles, we used a variety of quality assessment tools. We omitted 19 low-quality articles and kept eight for data extraction. Table 2 displays the characteristics of the eight studies included in this system review.

**Table 2:** Studies included in this review and its conclusion

Author	Journal and Publication year	Title	Conclusion	Type of research
Jan P A M Bogers [8].	Schizophrenia Bulletin Open, January 2020	Risk Factors for Psychotic Relapse After Dose Reduction or Discontinuation of Antipsychotics in Patients with Chronic Schizophrenia: A Systematic Review and Meta-analysis [8].	Dose reduction as low as possible	Meta-Analysis
Sandra Steingard [9].	Community Mental Health Journal, August 2018	Five Year Outcomes of Tapering Antipsychotic Drug Doses in a Community Mental Health Center [9].	Modest dose reduction	Observational Study
Jati Tiihonen [10].	The American journal of psychiatry, August 2018	20-Year Nationwide Follow-Up Study on Discontinuation of Antipsychotic Treatment in First-Episode Schizophrenia [10].	Continued for 1-5 years	Meta-Analysis
Mark Abie Horowitz [7].	Oxford Academic, July 2021	A Method for Tapering Antipsychotic Treatment That May Minimize the Risk of Relapse [7].	Precise percentage reduction/slow tapering	Analysis
Swapnil Gupta [11].	Cambridge University, August 2018	Deprescribing antipsychotics: a guide for clinicians [11].	Gradual deprescribing	Review
Stanley N. Caroff [12].	Boston Medical Center Psychiatry, July 2020	Hospital utilization rates following antipsychotic dose reduction in mood disorders: implications for treatment of tardive dyskinesia [12].	Maintenance treatment	Clinical study
Miriam L. Zachlin [13].	Springer open drug investigation, September, 2021	The Impact of Antipsychotic Dose Reduction on Clinical Outcomes and Health Care Resource Use Among Medicare Patients with Schizophrenia [13].	Need for alternative study, Increased ER visits after dose reduction	Clinical study/Drug investigation
Maximilian Huhn [14].	European archives of psychiatry and neuroscience, February, 2020	Reducing antipsychotic drugs in stable patients with chronic schizophrenia or schizoaffective disorder: a randomized controlled pilot trial [14].	Slow reduction of antipsychotics is feasible	Controlled trial

Discussion

Patient's Perspective on Taking Anti-Psychotics

In a study conducted, there were mixed opinions about taking antipsychotics; among them, one-third of individuals in this big poll indicated they were content with long-term antipsychotic treatment, while others reported that they took medicine unwillingly, accepted it temporarily, or actively opposed it [15]. A third of respondents expressed interest in attempting to discontinue medication with professional assistance, and almost half desired the option to reduce drug dosage. Many others were more hesitant but expressed a desire to reduce or cease medicine or do so in the future, in addition to having more negative attitudes toward medication, which is consistent with other studies on why patients cease antipsychotic treatment [15]. Several individuals claimed that the sole reason they took antipsychotics was that their physician advised them to do so [16]. Concerns about adverse effects, in particular sedative effects, neurological effects, and weight gain, as well as concerns about the impact of antipsychotics on physical health and functioning, were the driving force behind the desire to discontinue or minimize the use of medicine. These findings are similar to prior research on people's perspectives regarding antipsychotic medication [15,16]. Throughout the treatment, providers need to be responsive to changes in the patient's demands and treatment choices [9]. Particularly incorporating functional, social factors into the conversation, which has been demonstrated to be essential for remission and recovery in the past, appears to be essential for the development of meaningful discourse and the promotion of a successful outcome over the long term [9,15,17,18]. 

Maintenance Dose Recommended Protocol

Treatment for psychosis takes a longer time than for other health conditions. It may take years to fall into the category of remission. Treatment with antipsychotic medications for an extended period is connected with an enhanced chance of survival [10]. This might lead to tolerance and side effects, eventually leading to relapse [10]. Most schizophrenia guidelines support indefinite antipsychotic use after two psychotic episodes [19]. Current guidelines also suggest continuing antipsychotic medicine, with close monitoring for its efficacy and its adverse effects, and continued drug use for patients whose symptoms have shown significant improvement [11,19,20]. Furthermore, it also suggests several different therapy options available for the adverse effects of antipsychotic medication [16,17,18].

Maintenance Dose vs. Taper to a Minimum Effective Dose

Several counter-challenging researches and guidelines need extensive research in this sub-sector of the psychiatric division [21]. In patients diagnosed with persistent schizophrenia, we evaluated various studies that increase the likelihood of a psychotic episode after a decrease in antipsychotic drug dosage or its cessation [21,22]. The decision to continue maintenance dose treatment can be more straightforward if a guideline can be supported by the use of a symptom severity scale which can be made by including a correct definition and by making use of improvements such as a hormone level or imaging such as a PET scan [21,22]. A study conducted in Denmark advised that patients continue to receive maintenance treatment for at least one year following remission [23]. Consequently, patients in the maintenance group continue to get therapy as usual [13]. People with schizophrenia can have a worsening cognitive condition and an increase in the rate at which they are hospitalized if their antipsychotic dosage is decreased rapidly [13].

Withdrawal Syndrome

All psychiatric medication may cause withdrawal symptoms. Withdrawal symptoms are so widespread that they are an expected part of the pharmacology of any medication that is withdrawn faster than preexisting adaptations to the drug resolve. Receptor antagonists diminish target receptor activity, increasing receptor sensitivity, and physiological amounts of the receptor's ligand might overstimulate hypersensitive receptors, causing withdrawal or rebound symptoms. Abruptly stopping beta-blockers may trigger adrenergic rebound, including high blood pressure, heart rate, anxiety, headaches, and myocardial infarction [7]. After discontinuing antipsychotics, rebound symptoms, withdrawal symptoms, or developing new psychotic symptoms, such as disorderly conduct, might last longer than six weeks. When they stay longer than six weeks, they are chronic post-withdrawal illnesses [24,25].

Tapering Antipsychotics

Together with their patients, psychiatrists should gradually lower the antipsychotic dosage to the lowest level, which continues to be effective in preventing the recurrence of the patient's unpleasant symptoms [26]. It is possible to try gradually decreasing the antipsychotic medication that individuals with chronic stable schizophrenia use; this should be done cautiously and under constant monitoring [14]. In an examination of medical practice's deprescribing procedures, five critical components were identified: compiling a complete history of drugs, detecting potentially unsuitable medications, assessing if a potentially inappropriate prescription may be stopped, designing the withdrawal protocol (e.g., tapering when required), and providing monitoring, support, and documentation [11]. This five-step approach has been modified for use in psychiatry to incorporate proper scheduling, the patient's family, friends, and mental health team's participation in the deprescribing process, and formulating a strategy for relapse diagnosis, prevention, and treatment [11]. Antipsychotic deprescribing or tapering are not included in standard recommendations, while some guidelines advocate lowering to minimal effective dosages without describing how to do so [11,19,27,28]. The primary method for reducing the severity of withdrawal symptoms is to slow down the pace at which the equilibrium is not disrupted; This buys the body more time to undo the neuroadaptations by medication and go back to its original state [9,11].

Tapering may lessen the chance of experiencing withdrawal symptoms and the severity of those symptoms, including the possible risk of withdrawal psychosis [11]. After discontinuing antipsychotics, many patients have TD, the most apparent sign that dopaminergic hypersensitivity has set in, suggesting that adaptations to antipsychotics might take years to develop, indicating that extended tapering is necessary [12,29]. People who have been on these drugs for an extended length of time will likely need tapering periods that are at least as long to reduce the risk of psychotic recurrence [30].

As a direct therapeutic implication of our research, healthcare providers treating chronic patients with schizophrenia will need to give considerable thought before deciding whether or not to lower their antipsychotic medication dosage. If there is a need to lower the amount of antipsychotic medication, it is best to do so gradually to minimize the possibility of experiencing a relapse. It is not recommended to stop taking antipsychotic medication. However, during the first six months to a year following a dosage decrease, there is a need for increased guidance.

## Conclusions

The protocol recommended needs significant research to make changes and give proper guidelines in tapering antipsychotics. In the relevant literature, it is still unclear what defines the maintenance phase of psychosis and how it should be treated. This is because none of these issues has been adequately addressed. However, most of the studies suggest slow tapering aids in the reduction of relapse or withdrawal psychosis by reducing hospitalizations. In general, discontinuation and intermittent or targeted approaches are not recommended; nonetheless, there is significant controversy about discontinuing at a lower level, especially atypical antipsychotics. In general, cessation, as well as intermittent or targeted methods, is not recommended. It is maintained at the lowest possible dose after long tapering is suggested to a certain degree. Before strongly suggesting the cessation of antipsychotic medication at this crucial stage in treating schizophrenia, further research is required.
