# Correction: A Systematic Review of the Need for Guideline Recommendations; Slow Tapering vs. Maintenance Dose in Long-Term Antipsychotic Treatment: 2022

**DOI:** 10.7759/cureus.c350

**Published:** 2025-10-07

**Authors:** Shanthi Potla, Yousif Al Qabandi, Savitri Aninditha Nandula, Chinmayi Sree Boddepalli, Sai Dheeraj Gutlapalli, Vamsi Krishna Lavu, Rana Abdelwahab Mohamed Abdelwahab, Ruimin Huang, Pousette Hamid

**Affiliations:** 1 Psychiatry and Behavioral Sciences, California Institute of Behavioral Neurosciences & Psychology, Fairfield, USA; 2 Internal Medicine, California Institute of Behavioral Neurosciences & Psychology, Fairfield, USA; 3 Dermatology, California Institute of Behavioral Neurosciences & Psychology, Fairfield, USA; 4 Neurology, California Institute of Behavioral Neurosciences & Psychology, Fairfield, USA

This article has been corrected to fix the following typos in the text and PRISMA diagram:

- 'Records identified' corrected from 74 to 78
- 'Records screened' corrected from 56 to 60
- 'Reports assessed for eligibility' corrected from 23 to 27
- 'Reports excluded, low quality' corrected from 14 to 19

